# The Role of Proteins in Biosilicification

**DOI:** 10.6064/2012/867562

**Published:** 2012-10-01

**Authors:** Daniel Otzen

**Affiliations:** Interdisciplinary Nanoscience Center (iNANO), Center for Insoluble Protein Structures (inSPIN), and Department of Molecular Biology and Genetics, Aarhus University, Gustav Wieds Vej 14, 8000 Aarhus C, Denmark

## Abstract

Although the use of silicon dioxide (silica) as a constituent of living organisms is mainly restricted to diatoms and sponges, the ways in which this process is controlled by nature continue to inspire and fascinate. Both diatoms and sponges carry out biosilificiation using an organic matrix but they adopt very different strategies. Diatoms use small and heavily modified peptides called silaffins, where the most characteristic feature is a modulation of charge by attaching long chain polyamines (LCPAs) to lysine groups. Free LCPAs can also cooperate with silaffins. Sponges use the enzyme silicatein which is homologous to the cysteine protease cathepsin. Both classes of proteins form higher-order structures which act both as structural templates and mechanistic catalysts for the polycondensation reaction. In both cases, additional proteins are continuously being discovered which modulate the process further. This paper concentrates on the role of these proteins in the biosilification process as well as in various applications, highlighting areas where focus on specific protein properties may provide further insight. The field of biosilification is a crossroads of different disciplines, where insight into the energetics and mechanisms of molecular self-assembly combine with fundamental biology, complex multicomponent colloidal systems, and an impressive array of potential technological applications.

## 1. Introduction

**Figure 1:**
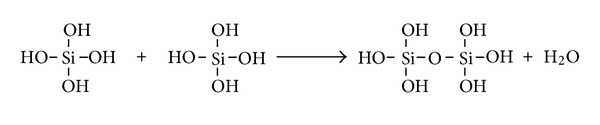
Condensation of two orthosilicate molecules with the elimination of water. This process can be continued *ad infinitum *to lead to long insoluble biosilica molecules.

Biomineralization is the formation of complexes containing inorganic materials by living organisms. This occurs in organs as diverse as bone, teeth, egg shells, and invertebrate exoskeletons [1]. Calcium is a very “popular” biomineral, occurring for example, as phosphates in vertebrate skeletons and carbonates in mollusk shells. However, another important player is silicon. Silicon is the second most common element in the Earth's crust after oxygen, and silica (silicon dioxide) is the most abundant compound in the earth's crust. Biosilicification is the process by which inorganic silicon is incorporated into living organisms as silica, which occurs on the scale of gigatons [2]. In practice this involves the condensation of orthosilicate Si(OH)_4_ into long polymers with the elimination of water (Figure 1). The process mainly occurs in the unicellular diatoms and the multicellular sponges, but silica also deposits in plants [3] and even in higher mammals, having been reported in the electric organs of the fish *Psammobatis extenta* [4]. 

**Figure 2:**
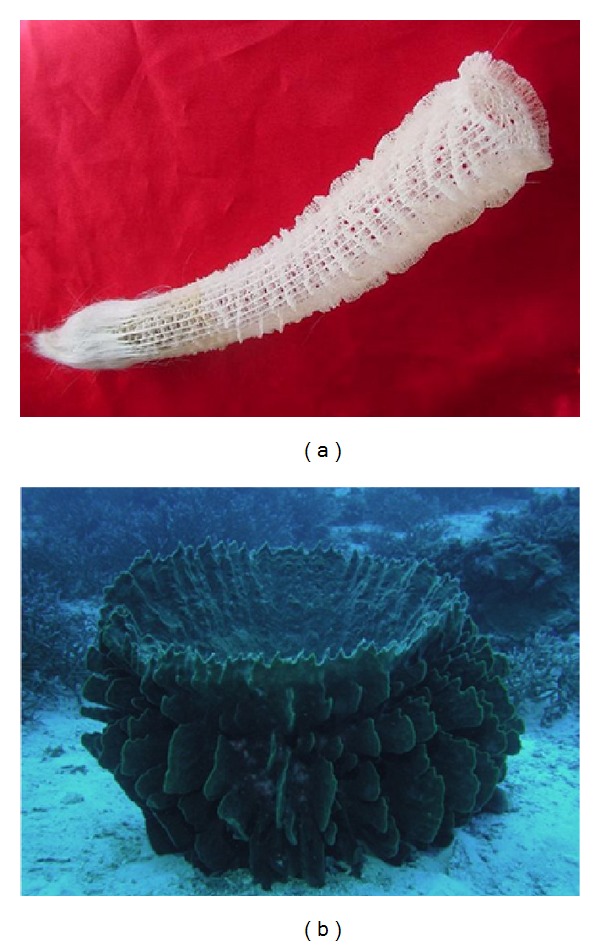
Siliceous sponges. (a) Venus' Flower Basket (*Euplectella aspergillum*), greatest dimension 25 cm (hexactinellid). Copyright Heidi Reed. (b) Barrel sponge (*Xestospongia testudinaria*), demosponge.

**Figure 3:**
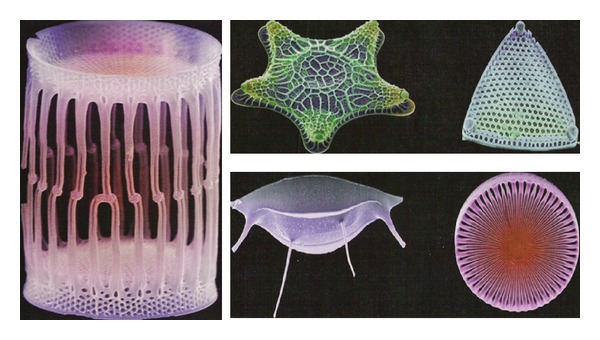
Images of various diatoms. Reproduced courtesy of Mark Hildebrand and New Scientist, 2004.

The deposition of silica is fascinating in many ways. At the purely visual level, silicification leads to an exquisitely beautiful outcome. Right from their first descriptions, uni- and multicellular silicifying organisms drew admiring comments; they were considered to be without “rival [s] in beauty” [5, 6] (Figure 2). High magnification images of the cell walls of diatoms continue to amaze with their intricate and seemingly delicate structures and infinite variety of nanostructures [7] (Figure 3). What is particularly amazing is that this process occurs under physiological conditions at temperatures between 0 and 37°C, neutral pH, and ambient pressure, and biosilicification is around 10^6^ times faster than the corresponding abiotic process [8]. In contrast, chemical synthesis of materials containing silica (typically used as semiconductors, chromatographic resins, ceramics, plastics, and insulators) proceeds at extreme values of pressure, pH, and temperature. Yet the biosilica formed by humble diatoms and sponges is no less robust than synthetic glass; it only melts at temperatures above 2000°C [9] and shows high material toughness, strength, and elasticity [10] as well as impressive light-transmitting properties [11]. The secret behind these marvels lies in the integration of the silica material into an organic matrix consisting of proteins and in some cases organic molecules such as long chain polyamines (LCPAs). This matrix sculpts the silica to form nanoscale composites under exquisite morphological control. Since the first identification of silica-forming proteins around 1998-1999 there has been immense progress in the understanding of the role which proteins and LCPAs play in the process of biosilicification, yet many questions remain unanswered both at the level of overall architecture and the molecular details of the outcome. The key issue is how the organic components sculpt an inorganic process into a biological structure with a well-defined function.

This paper will try to address the process of biosilification from the perspective of the proteins involved. There are many truly excellent reviews on the topic of biosilicification, though they tend to concentrate either on diatoms [12, 13] or sponges [14–22], with only a few treating both [23]. Here I will compare the two major classes of biosilification, trying to highlight common principles, and the ways in which protein science may contribute to further advance this field.

## 2. The Two Classes of Silicifying Organisms

The unicellular algae termed diatoms contain a silica cell wall (frustule) which consists of two overlapping valves with interconnecting segments or girdle bands. Valves are formed during cell division within a few minutes by controlled precipitation of silica. Silica is concentrated within the silica deposition vesicle which is encased by the silicalemma membrane. When the siliceous wall in diatoms has matured, it is expelled and a new plasmalemma is formed underneath it [24]. The valve structure is a hierarchy of self-similar or fractal patterns. Within each valve, each hexagonally arranged chamber (areolae) contains a set of hexagonally arranged pores (cribrum) which in turn embodies a set of even smaller hexagonally arranged pores (cribellum). Cell wall maturation occurs in a centripetal or top-down fashion, in which first the areolae form, followed by cribrum, and then cribellum [25].

**Figure 4:**
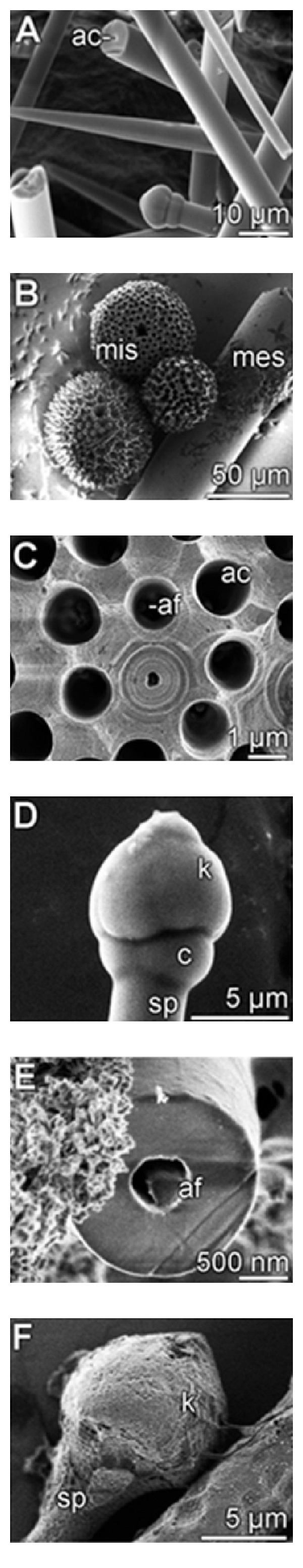
Spicules of (A, D–F) *S. domuncula* and (B, C) *G. cydonium*. (A) The skeletal tissue of *S. domuncula* only forms long spicules called megascleres which are either tylostyles (spicules with swelling at one end) or styles (pointed at both ends); the axial canal <(ac) visible where one spicule is broken. (B) Microscleres (mis) are composed both of megascleres (mes) and microscleres, consisting of many thin rays radiating from a globular center and with the axial canal clearly visible in cross sections (C), sometimes with the axial filament (af) visible. (D) The tylostyle (sp) swelling has a terminal knob (k) atop a collar. (E) The axial filament may be seen more clearly in a spicular cross section. (F) The underlying axial filament is more clearly seen when the spicule is partially dissolved in hydrogen fluoride. Reproduced with permission from [38].

The diatoms' cell walls, within which the silica is integrated, are no bigger than a few *μ*m. In contrast, sponges form cylindrical siliceous structures known as spicules which can be up to 3 m long and several cm wide as in the case of *Monorhaphis chuni*. There are two classes of sponges within the phylum Porifera which produce siliceous spicules, namely, the Demosponges and the Hexactinellids. They differ in the structures of the spicules, which tend to only have one or four axes in the Demosponges (Figure 4) and six axes for the Hexactinellids. Spicules are formed within sclerocyte cells [26]. Within the Demosponges, the spicules tend to fall into different size classes. Megascleres, with lengths beyond 300 *μ*m, constitute the bulk of the sponge skeleton while microscleres are much smaller and more variable in size and shape and have ancillary function [27] (Figure 4). In both sponge classes, the spicules contain a core protein structure, the axial filament [28], and silica deposits around this filament in an intricate periodic arrangement, leading to concentric layers or lamellae. These lamellae fuse or biosinter in demosponges upon maturation but remain more distinctly lamellar in hexactinellids. Silica is stored within intracellular granules called silicasomes [29] which release their content extracellularly by exocytosis [30], transferring silica to the lamellae.

## 3. Uptake of Silica: Membrane-Bound Silicon Transporters with Silicate-Binding Motifs

**Figure 5:**
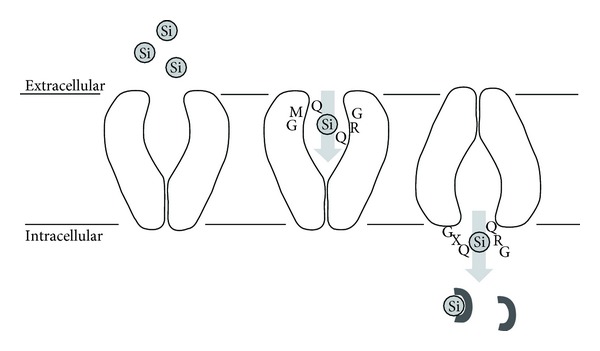
Proposed model of silicon transport through the 10-transmembrane diatom transporter. The outward-facing conformation binds extracelllar silicate through hydrogen bonding to 2 conserved Gln in the transmembrane helices 7 and 8. A conformational change to an inward-facing conformation allows silicate to bind to other conserved Gln in the loop between helices 2 and 3, releasing silicate into the cell to be bound by as yet unknown components. Reproduced with permission from [36].

Proteins have to come to the rescue already at the very first stage of silicification, namely, the accumulation of silicate within cells to concentrations high enough for subsequent deposition. The concentration of silicic acid is around 3–70 *μ*M in seawater, depending on the biological consumption of silica in the local environment [2]. This is actively transported into the cell by silicon transporters, leading to intracellular concentrations above 100 mM. This concentration is so high that silicate is effectively supersaturated, providing a clear indication that other components such as proteins have to be present to maintain the silica in a soluble state. Because of the low concentration of silicic acid outside the cell, silicon uptake requires a specialized active transport system. Only one transporter has so far been identified in sponges, namely, a protein in *S. domuncula* assigned to the NBC transporter family, whose expression is upregulated by increasing the silicic acid concentration and whose activity is localized to areas close to spicules [31]. This transporter is predicted to contain 10 transmembrane helices in the C-terminal region and to cotransport sodium ions with silicate ions, but no silicate binding motifs have been identified. In contrast, numerous transporters are known from diatoms, starting from the first description in *Cylindrotheca fusiformis* using the Si analogue ^68^Ge as tracer [32]. These are also integral plasma membrane proteins [33] also with 10 predicted and highly conserved transmembrane helices [34], which work as silicate/sodium symporters in 1 : 1 transport stoichiometry [32] and with apparent protein-silicon interaction constants of moderate affinity (0.5–10 *μ*M). These affinities lie in a concentration range slightly below that of silicate concentration in sea water [35]. The less conserved C-terminal domain of the transporters may interact with other proteins. The sodium binding sites are similar to those of other sodium transporters, while silicate is proposed to be taken up and transported over the membrane via hydrogen bonding to Gln residues in GXQ motifs [36] (Figure 5). Transporter expression is induced prior to maximal silicon uptake and before cell wall silicification starts [37].

### 3.1. Challenging Issues: Structure and Protein Engineering of Silicate Transporters

Obviously the transporters' silicate binding motifs are likely to be different from the motifs promoting binding to proteins for condensation reactions, since the silicate only has to be bound transiently without (presumably) undergoing any chemical changes (it has to be said that we still do not know what chemical species is the precursor to the final silica structure, see below). Nevertheless, it will be very interesting to obtain more structural information about this class of proteins and contrast their silicate binding/release strategy with that of silicate condensation proteins. Although no high resolution structure is available of silicate transporters, it has recently become possible to express the transporter SIT3 from the diatom *T. pseudonana* in *S. cerevisiae* [39], based on GFP fusions to optimize expression and purification. The protein is active in reconstituted proteoliposomes and forms a homotetrameric *α*-helical membrane protein. Its low affinity (K_0.5_ ~ 20 *μ*M) suggests that it may be a member of an “early warning system”; at low silica concentrations, the binding sites will not be filled to the same extent, possibly leading to the upregulation of high-affinity transporters. There are also silicon transporters in rice [40] and other plants [41], but they are not homologous to the diatom transporters and may use a different transport strategy altogether. Much information may be obtained by further analysis of silicon transporters, particularly in terms of atomic level structure. Silicate transporters constitute well-defined protein entities firmly embedded in the plasma membrane and there should not be any methodological barriers to their crystallization, apart from the usual experimental challenges for crystallization of membrane proteins such as obtaining mg amounts of pure, monodisperse, and active transporters which can crystallize in an appropriate detergent/lipidic matrix (see e.g., [42] and related articles in that issue). Additional information may be obtained by selection for transporter mutants with increased uptake of silicate.

## 4. Identifying Proteins Involved in Biosilicification

There are two major strategies to identify proteins involved in biosilicification: the direct and the indirect approach. In the direct approach, proteins are directly isolated from the silica of biological specimens, that is, either the mature cell wall of the diatom or sponge spicules. Their biosilicifying activity may be determined by their ability to form precipitates of silica from solutions of either silicic acid (diatom proteins) or organic precursors such as tetraethoxysilane (sponge proteins); the pelleted silica may then be hydrolysed in alkali and its concentration measured using the colorimetric molybdic blue assay [43, 44]. Indirect approaches are typically global analyses such as whole genome expression profiling which may compare the levels of different RNA transcripts produced under conditions where silicate is limited versus in excess [45] and correlate whose genes' transcription correlates with that of known silicifying proteins [46]. Candidate genes may in both cases subsequently be corroborated by immunostaining or coexpression with reporter genes such as GFP. The two approaches may also be combined in yeast two hybrid screens [38, 47] or pull-down assays [38] where proteins linked to biosilicification are used as bait.

Given that biosilica structures are usually very robust and resistant to most chemical and physical insults, it is challenging to extract proteins from these composites without degrading or chemically modifying them. Since the first reports of silicifying proteins in the late 1990's, more proteins have been discovered—and the understanding of the chemical nature of the proteins involved has advanced—as extraction methods have become more exhaustive and also less chemically aggressive, and it is likely that additional proteins will be added to the list in the coming years. This will be aided by the steady increase in the number of genomes from different silicifying organism [49]. Nevertheless, the chemical modifications of different silicifying proteins and the role of nonproteinaceous components such as long chain polyamines continue to make elucidation of biosilicification strategies challenging at both the chemical, biological, and biophysical level.

### 4.1. Diatoms: Silaffins and Friends

Sumper and coworkers were the first to identify proteins directly involved in diatom silicification. In a landmark paper in Science in 1999 [50], they first removed relatively loosely bound proteins from the isolated cell wall of the diatom *Cylindrotheca fusiformis* using chemical procedures such as EDTA. They then used anhydrous hydrogen fluoride (HF) to dissolve the silica structure and leave adhering proteins behind which were separated by cation exchange chromatography, exploiting the highly cationic nature of some of these proteins (see below). This process identified two classes of proteins: the first class is a class of proteins around 200 kDa called HEP200 (HF-extractable proteins) or pleuralins [51], which are tightly bound to the silica via silica strips which braid the cell and are shielded by more peripherally bound Ca^2+^-binding proteins (frustulins) [52]; none of these proteins are actively involved in biosilicification. The second and more abundant class involved a band of proteins around 4–17 kDa, called *silaffins*. Of these, silaffin-1A (in itself a mixture of peptides) migrated around 4 kDa, silaffin-1B around 8 kDa, and silaffin-2 around 17 kDa. All these proteins were individually able to precipitate silica as nm-sized spheres from a metastable solution of silicic acid within seconds. A key to the polycondensation properties of these proteins lies in their chemical structure. The silaffin-1 peptides derive from a 265-residue sil1p polypeptide, of which residues 20–107 contain many acidic residues while residues 108–265 are more basic, containing Lys-Lys and Arg-Arg motifs in a repetitive structure (repeats R1–R7 of sizes 19, 22, or 33 residues). The peptides identified in the silaffin-1A_1_ band are around 2.5–3 kDa and represents individual repeats R3–R7, cleaved at sequence motifs RIL or RNL, while repeats R1 and R2 give rise to silaffin-1B and silaffin-1A_2_, respectively. In the diatom *Thalassiosira pseudonana*, another intensely studied diatom, the identified silaffins tpsil1-3 [53] show no sequence homology with* C. fusiformis* silaffins but have the same general amino acid composition and post-translational modification (see below). These silaffins are derived from three precursor polypeptides which are cleaved in different ways; only the low-molecular forms form porous silica *in vitro*.

**Figure 6:**
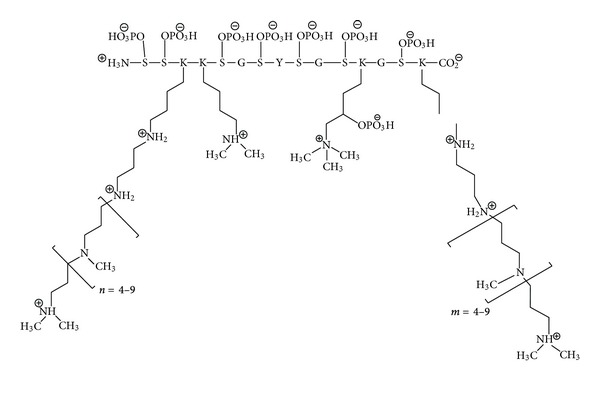
Chemical structure of silaffin-1A1. The lysine modifications include oligo-N-methyl-propylamine, *ε*-N,N-dimethyl-lysine, and *ε*-N,N,N-trimethyl-*δ*-phospholysine. There are 7 serine phosphorylations. Reproduced with permission from [23], adapted in turn from [48].

The 19-residue peptide repeat R5 (SSKKS GSYSG SKGSK RRIL) has subsequently been used in numerous attempts to harness silicification *in vitro*. Its C-terminal RRIL motif is critical for activity [55]. Synthetically prepared R5 is unable to precipitate silica below pH 7, whereas native silaffin-1A has maximal activity at pH 5 and persists down to pH 4 [50]. This low-pH activity nicely reflects the acidic environment of the silicon deposition vesicle in which silica formation takes place [56], and derives from an unusual property of the silaffins, namely, covalent modification. Modification occurs in two different ways:

(a) *Lys modification with polyamines and methyl groups:* silaffins contain numerous Lys-Lys pairs, in which the first Lys is typically linked to 6–11 repeats of N-methyl-propylamine or another long-chain polyamine, while the second Lys is modified to either *ε*-N,N-dimethyl lysine or *ε*-N,N,N-trimethyl-*δ*-hydroxylysine [48] (Figure 6). Such a modification allows for a combination of cationic and hydrogen-bonding interactions to bind tightly to the surfaces of silica particles. In *C. fusiformis*, the polyamines are all attached to the silaffin backbone, but in other diatoms they also exist as free long chain polyamines (LCPAs) and are intimately involved in biosilicification [57]. Each LCPA is typically 0.6–1.5 kDa long and is based on a (usually N-methylated) propylamine, usually attached to ornithine or its decarboxylated product putrescine with different degrees of N-methylation. A more detailed empirical rule has been formulated based on the modified lysines of silaffin-3 from the diatom *Thalassiosira pseudonana*, where 30 of the 33 Lys are found in a K-A/S/Q-K tetrapeptide. Here the first Lys has two aminopropyl units (4,8-diazaoctanyl-residue) while the second lysine (if separated by at least 5 aa from other tetrapeptide clusters) is an *ε*-N,N-dimethyllysine; with shorter separations, both subsequent K are modified by two aminopropyl units [58]. Clearly there has to be a sophisticated multistep enzymatic machinery for these modifications but the details are only beginning to be elucidated [59]. 

(b) *Ser phosphorylation:* if hydrogen fluoride extraction is replaced by gentler ammonium fluoride treatment at pH 5, protein-phosphate ester bonds will not be hydrolyzed. This allowed Sumper and coworkers to identify 8 phosphate groups linked to silaffin-1A [60], of which 7 bind Ser and 1 binds a trimethyl-hydroxyl-Lys (Figure 6). These phosphate groups affect SDS-PAGE significantly, increasing the apparent molecular weight from ~4 to 6.5 kDa The presence of phosphate groups makes silaffin 1A able to precipitate silica in the absence of phosphate buffer. If the phosphate group is not present on the protein, it has to be supplied in buffer form and is used up stoichiometrically in the process. Silaffin-2 and silaffin-1B in *C. fusiformis *are also phosphorylated to significant extents [61]. In addition to LCPAs and phosphate groups, silaffin-2 is modified by hydroxyproline, sulfates, and complex carbohydrates, and all in all possesses an exceptionally high negative charge density. This high anionic density means that native silaffin-2 cannot (but deglycosylated and disulfated silaffin-2 can) precipitate silica *in vitro* unless helped by LCPAs, and that native silaffin-2 actually inhibits native silaffin-1A's silicification activity at high silaffin-2 concentrations, probably by shielding silaffin-1A's positive charge [61]. Glycosylation also plays a role in other silaffins; for example, the high molecular weight forms of silaffins from *T. pseudonana* are highly glycosylated as well as containing dihydroxyproline, and they are unable to precipitate silica *in vitro *[53]. Another phosphorylated protein from *T. pseudonana* is silacidin, a highly acidic low-molecular weight peptide which mainly consist of Ser (more than 60% of which is phosphorylated), Asp and Glu [62] and is thus likely to be mainly unstructured. Silacidin promotes silica precipitation in the presence of LCPA, perhaps by forming large-scale structures due to electrostatic interactions, and thus possibly working as a “first-aid agent” ensuring effective silica processing in silicic acid depleted habitats [63]. A membrane-associated Ser-specific silaffin kinase has recently been identified in *T. pseudonana *based on its similar expression pattern as tpsil3 [64]. However, it only phosphorylates a fraction of all silaffins and accounts for only ~25% of all silaffin kinase activity, indicating that many other kinases are active. 

#### 4.1.1. How Is Silica Precipitation Regulated by the Active Components?

**Figure 7:**
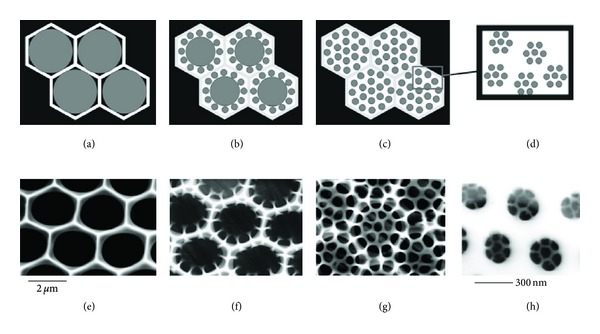
Templating of diatom cell wall structure by the physical-chemical properties of long-chain polyamines within the silica-deposition vesicle in the phase separation model. (a)–(d) are models and (e)–(h) are scanning electron micrographs of the silaffin-free diatom *C. wailesii* valves at different stages of growth. (a) A monolayer of LCPA droplets in a hexagonal arrangement. (b) and (c): Stepwise segregation into smaller droplets provides new locations for silica precipitation to the final dispersion into 50-nm droplets. Silica precipitation takes place within the water phase (white areas). Reproduced with permission from [54].

Silaffins have no monopoly on silicification. Even simple lysine oligomers can stimulate silica precipitation *in vitro* in a way that increases strongly with peptide length [65], and the very basic enzyme hen egg white lysozyme also stimulates silica precipitation in an act of self-encapsulation [66]. Dendrimers terminated with polyprolyeneimine also form silica nanospheres with diameters of 170–400 nm, probably because positive patches on the dendrimer surface bind silanoate or silica species [67]. In fact, LCPAs in *Coscinodiscus *(which has no silaffins) have been proposed to be intrinsically able to drive silica precipitation and pattern formation in an ingenious model proposed by Sumper [54], in which the amphiphilic nature of the LCPAs leads to repeated phase separation within the silicon deposition vesicles in a hierarchical fashion, ultimately fashioning the areolae-cribrum-cribellum arrangement in a top-down approach until all the LCPA is used up (Figure 7). Microemulsion formation will be stabilized by increased methylation [68] while charged and uncharged amino groups may provide a pH-switch; partial protonation of LCPAs at neutral pH enables them to act as Brønsted acids to promote silica condensation [69]. Further modulation is provided by variations in the intramolecular amine-amine spacing and the carbon:nitrogen ratio [70]. However, phosphates are also required for LCPA silica precipitation through microscopic phase separation via the formation of a hydrogen-bonding network stabilized by electrostatic interactions [71], and this illustrates how phosphorylated silaffins can profoundly modulate this process. Similarly, phosphate groups are required for nonsilaffin peptides to induce silica condensation [72, 73] by nucleating peptides around the phosphate group.

Fundamentally, the activation barriers to silica condensation include the diffusion-limited collision of silicate particles and the accumulation of negative charge in the consequent oligomers. Peptides and LCPAs can reduce this barrier by increasing the local concentration of silicate particles (as nucleation points or scaffolds, as suggested by simulation studies [74]) and complementing the charge accumulation, both through electrostatic interactions. LCPAs and silaffins in *C. fusiformis *can precipitate silica alone and together but with different outcomes. In *T. pseudonana* at acidic pH (5.5), different silaffins only precipitate silica together with LCPA and with different concentration dependencies and morphologies (ranging from spheres of different sizes to porous sheets and plates) [53]. Simple inhibition of the ornithine decarboxylase in *T. pseudonana*, which reduces the ability to form LCPAs, leads to a drastic alteration of the silica structure [46]. The obvious corollary is that different combinations of silaffins and LCPAs can govern the different morphologies of diatoms. 

#### 4.1.2. Challenging Issues: The Interplay between Silaffins and LCPA in Self-Assembly and Silicification; Small Angle Scattering Techniques with Reconstituted Complexes

There is likely to be a very complex interplay between the different components of silaffins, their post-translational modifications and LCPAs in their self-assembly and organization of the diatom cell wall. Given that there are so many different ways of precipitating silica with different combinations of silaffins and LCPAs where the modifications on the silaffins also will affect the outcome, it is probably going to be difficult to establish a single unambiguous pathway *in vivo*. A major goal of biophysical studies is to establish rules or principles for how this may occur. Despite the enormous variety of silaffins and LCPAs, there may be simple guidelines at play. There are already tantalizing hints. Native silaffin-1A self-assembles to large aggregates at low ionic strength (as measured by ^31^P-NMR line broadening) [60], just as it is able to form spherical clusters, strand networks and bicontinuous structures in coarse-grained simulations [74]. Furthermore, the LCPA modifications of native silaffin-2 may mediate its self-assembly [61]. Native silaffin-1A and native silaffin-2A coaggregate at low ionic strength and can be pelleted, unlike the individual peptides [61]; different ratios of the two peptides lead to different morphologies. Silaffin-2 can be seen as a polyanionic regulator of silica formation, much as surfactants can modulate the effect of block copolymers on the synthesis of mesoporous silica [61]. Similarly, hydroxyl-containing molecules such as alcohols and carbohydrates dramatically decrease the sizes of silica particles formed by R5 peptides, probably by reducing the nucleation barrier by providing additional hydrogen-bonding partners [75]. Clearly it will be necessary to work out in greater detail how these aggregation steps occur, while taking into account the constraints provided by the biological environment, for example, the dynamics of microtubules and actin which may control the micro- and mesoscale positioning of silica structures [76].

Detailed information about the molecular interactions between silicate and peptides/LCPAs leading to silica condensation will be a great help. Atomic-level structures by X-ray crystallography are not going to be easy to obtain here because the diffuse and colloidal nature of the different silaffin-LCPA complexes are an obstacle to the precise lattice contacts required for crystallization; they are also likely to be too large and polydisperse for solution NMR structures. However, *in situ* Si-NMR studies of the chemical changes to silicate ions and their local environment may provide a certain level of insight, either *in vitro* or *in vivo*. Another promising approach would be to carry out a systematic analysis of the assembly behavior of different silaffins alone and together with LCPAs using low-resolution techniques such as small angle X-ray scattering which can follow the structural evolution of complex assemblies of for example, proteins alone and in combination with other self-organizing molecules such as surfactants in real time [78, 79]. It will be a challenge to obtain sufficient amounts of authentic protein, though reconstruction of the enzymatic machinery, which has recently started to be dissected [59], may help. Perhaps transfer and whole-sale recombinant coexpression of the involved enzymes (bacterially derived polyamine synthesizing S-adenosylmethionine decarboxylase (AdoMetDC) and an aminopropyltransferase, sometimes fused to a eukaryotic histone N-methyltransferase domain, that potentially synthesize and N-methylate LCPAs) may allow us to reconstitute a high-yielding modification system. 

We can also expect other proteins to be discovered which will further nuance the overall picture. A very recent example includes the cingulins, obtained from a bioinformatics mining of the *T. pseudonana* genome for silaffin-like proteins (i.e., proteins with domains of at least 100 residues with at least 18% Ser and 10% Lys as well as an ER signal peptide). Of the 89 peptides found, 6 contained highly repetitive domains, where silaffin-like regions (KXXK) alternative with Trp- or Tyr-rich domains [80]. These proteins at located at girdle bands and bound so strongly that they are not even released by anhydrous hydrogen fluoride, hence precluding their identification by conventional means. Perhaps they represent the inner core of the scaffold that aggregate via hydrophobic or aromatic interactions of the Trp/Tyr rich domains? It is intriguing that these proteins may be just as vital as silaffins in the silicification process.

### 4.2. Sponges: Silicatein, the Glassy Version of Cathepsin

Given that biosilicification by all accounts has arisen independently in diatoms and sponges, it must come as no surprise that there are significant differences between the silica deposition strategies—and the outcomes of the process—in these two groups. The major differences can be encapsulated in Table 1.

As was the case for the diatom silaffins, the major silicifying component of sponges was identified by direct protein analysis of sponge material [81]. The sponge *Tethya aurantia* was bleached and soaked in acid, leaving mainly the needle-like skeletal elements called spicules (diameter ~30 *μ*m, constituting 75% of total sponge dry weight) from which the silica was removed by anhydrous hydrogen fluoride. The remaining material are fibril-shaped axial filaments (diameter ~2 *μ*m, only 0.1% of total spicule weight) gave 3 SDS-PAGE bands, identified as silicateins (silica proteins) *α*, *β*, and *γ*; these are present in the ratio 12 : 6 : 1 in *T. aurantia* which may reflect a specific structural arrangement involving these three peptides. Cloning of the major 330-residue protein silicatein *α* showed the protein to be 45% identical to the lysosomal cysteine protease cathepsin (including full conservation of 3 disulfide bonds), but with the major difference that cathepsin's catalytic Cys is replaced by a Ser in silicatein, while the other two members of the catalytic triad are in both cases His and Asn. (there may be silicateins with catalytic Cys instead of Ser [82]). The axial filaments survive the harsh extraction procedure (though they can be denatured by boiling [83]) and are able to catalyze formation of insoluble silica from organosilanes like tetraethoxysilane (which is otherwise stable in water), leading to precipitation along the filaments' long axis and ^29^Si-NMR evidence for an incompletely polymerized silica network [83]. Silicatein-like proteins have also been discovered in many other sponges [84] including proteins in the ratio 4 silicatein *α* : 1 silicatein *β* in *Suberitus domuncula* [85], a dimerizing silicatein in *Petrosia ficiformis* corresponding to silicatein *β* [86], and even silicateins from nonspicule forming sponges [87]. Many of these silicatein-producing sponges come from Lake Baikal which continues to be a rich source of diversity amongst the siliceous sponges [88, 89].

Silicatein is a many-faceted enzyme with multiple activities and self-association patterns. The precursor protein is 35 kDa large (including a 15-residue signal peptide), but in addition to the signal peptide, there is a 87-residue propeptide which is removed autocatalytically by an as yet unidentified mechanism [90], leaving a 23 kDa mature protein (this property likely explains its reported proteolytic activity in some contexts [91, 92]). Silicatein's silica-related enzymatic activity has prompted many investigations. No cofactors are involved and the process can proceed in simple buffer systems although Fe^3+^ has been reported to promote the reaction [93], while Mg^2+^ and EDTA have no effect. Mutation of the two major catalytic residues Ser 26 and His165 in *T. aurantia* reduces activity 10-fold to a level which is still twice that of the denatured protein [94]. While it is generally agreed that the mechanism requires a nucleophilic Ser group whose H-bond to His' imidazole group enhances the efficiency of the S_N_2 attack, the details of the enzymatic mechanism remains controversial. Silicon ethoxide condensation has been proposed to occur via a Ser-Si covalent intermediate [83], but this has not been demonstrated directly though a very thorough model has recently been proposed [95]. It has not been possible to crystallize silicatein due to its tendency to aggregate to higher-order structures, but it is possible to crystallize and determine the structures of mutants of cathepsin with the ability to condense silica from silicic acid [96]. In these mutants, mutation of cathepsin's active site Cys and the two adjoining residues to the corresponding silicatein mutants was sufficient to induce this activity. The authors propose a simple deprotonation reaction in which His163 deprotonates Si(OH)_4_ to form a template for subsequent condensation without a covalent intermediate. Confusingly, these constructs show no activity against silicatein's “normal” substrate TEOS, and this makes it more doubtful whether the cathepsin chimeras are appropriate model systems. 

Silicatein mimetics exploit small molecule compounds to mimic the nucleophile-hydrogen bond acceptor Ser-His arrangement separated by a suitable carbon spacer, for example cysteamine (SH nucleophile and NH_2_ acceptor) and ethanolamine. This can lead to particles of size 40–100 nm based on organosilane precursors [97]. Analogously, block copolymers of Cys/Ser and Lys also make silica deposits from TEOS, leading to SiO_2_ spheres (with reduced Cys) or blocks (with oxidized Cys) [98]. More spatially controlled deposits can be formed out by mixing two populations of gold particles modified with imidazole and hydroxyl groups, respectively [99].

Interestingly, in addition to its condensing or anabolic activity as a silica polymerase, silicatein may also have catabolic activity, given that it is able to cleave bis-*p*-aminophenoxy-dimethylsilane as a silica esterase [30], and it is even speculated that the switch from one activity to the other is controlled by low molecular weight compounds [30]. Perhaps such compounds could be present in silicasomes, where monomeric silicic acid may self-condense and could be hydrolysed back by silicatein. Alternatively, depolymerization activity may be taken care of by the 43-kDa silicase [101] discovered in *S. domuncula*, which is related to carbonic anhydrases. Just as the anhydrases control the hydration of carbon dioxide, silicase can dissolve biosilica and is upregulated at higher orthosilicate concentrations, perhaps to allow for a more dynamic equilibrium between mineralized and soluble silica.

#### 4.2.1. Silicatein: An Enzyme for All Elements?

Silicatein is not specific for silica precursors. *T. aurantia *silicatein it also acts on related metal oxides such as gallium nitrate [102], leading to deposition of gallium oxyhydroxide [103]. Alkoxides of titanium can be transformed to titanium oxide nanocrystals called anatase [104] and the barium salt BaTiF_6_ forms nanocrystalline BaTiOF_4_ with crystalline floret microstructures [105]. Layered nanoparticles of zirconia (ZrO_2_) can also form with appropriate Zr-containing precursors [106]. This is remarkable given the large variation in coordination numbers for these metals, varying from 4 for Si over 6 for Ti to 6–8 for Zr. In all cases, silicatein produces these polycondensations on stable and water-soluble precursors under mild temperature and pH conditions. Silicatein also reduces the gold salt HAuCl_4_ to anisotropic gold nanocrystals when the enzyme is immobilized on modified TiO_2_ nanowires [107]. Even more spectacularly, silicatein can catalyze the polymerization of a completely unrelated precursor, L-lactide, to poly(L-lactide) through a ring-opening step [108], though the mechanism behind this and its possible link to its autoprocessing proteolytic activities [90] remain unclear. These activities are not limited to *in vitro *conditions. When primmorphs (aggregates of proliferating cells formed from dissociated sponge cells, in this case *S. domuncula*) are grown in the presence of a TiO_2_ precursor, the TiO_2_ ends up integrated with SiO_2_ into the spicules [109]. All these observations make it paramount to obtain detailed atomic-level structures of silicatein in complex with silica precursors, but this is hampered by its built-in tendency to aggregate (see below). Perhaps clever strategies to reduce this self-aggregation property (by judicious mutation of selected “sticky” residues on the protein surface to polar or charged residues) may lead to stably monomeric silicatein that can be crystallized.

The activity of silicatein may not be confined to the active site alone, however. Compared to cathepsin, silicatein has fewer charged amino acids and more hydroxylated amino acids [81], including various Ser clusters [111]. A very recent study elegantly shuffled genes for silicatein *α* and *β* in *T. aurantia *in combination with random mutations in single-bead microcompartments, using bead sorting to select for variants with increased silica depositioning activity [112]. The mutants with increased activity had an increase in hydroxylated amino acids or mutations close to hydroxylated amino acids, highlighting the role of this class of residues, perhaps via hydrogen bonding interactions with silica precursors and/or the resulting silica structures. Silanol OH groups are known to be closely involved in hydrogen bonding with proteins [113].

#### 4.2.2. Self-Assembly of Silicatein *In Vitro*

Whatever the mechanism of silica deposition in sponges, there is no doubt that silicatein plays a central role in the process. Accordingly, its spatial distribution as well as its conformation in the spicule structure has been under intense scrutiny. The secondary structure of silicatein in spicules is dominated by *β*-sheets [113], in contrast to cathepsin, which consists of 31%  *α*-helix and only 20%  *β*-sheets [114]. Assuming that monomeric silicatein has the same overall structure as cathepsin, silicatein must undergo a major conformational rearrangement when it associates. Furthermore, this rearrangement and subsequent packing of the silicatein units may vary from one silicatein to another according to fiber diffraction studies on spicules from different species [113]. A model has been proposed for such a rearrangement at the monomeric level based on classical denaturation studies of a cathepsin-silicatein hybrid previously used for crystallization studies [96]. This protein undergoes two transitions in chemical denaturants, leading to the loss of tertiary and secondary structure, respectively; of more interest is the fact that the thermally denatured species is rich in *β*-sheet and may resemble the species that according to neutron reflectivity adsorbs to silica beads to form a stable partially unfolded monolayer [115]. In this form the positively charged residues may mediate binding to the silica while the active site as well as the Ser cluster are exposed for further catalysis.

The conformational transitions of proper silicatein may be more complicated than this, however. For one thing, the extraction method determines the level of association of silicatein and careful attention should be paid to this in any thorough study of silicatein self-association. The conventional but harsh hydrogen fluoride extraction—which still yields silicatein able to condense organosilane precursors—leads to formation of bands of 25, 50, 75, and ca. 96 kDa for axial filaments from *S. domuncula*, corresponding to silicatein monomers-tetramers, while extraction with Tris and glycerol only leads to monomer bands and a better separation of the *α* and *β* forms [91]; furthermore, the monomer from this preparation migrates as an 18 kDa band in the absence of reducing agent, only forming the (denatured) 25 kDa band when reduced. Remarkably, the glycerol-extracted silicatein, but not its HF-extracted counterpart, showed proteolytic activity in a simple casein assay (though the silicification activity was unfortunately not reported). If allowed to reassemble, Tris-extracted native silicatein forms long (100–150 *μ*m) filaments with regular branching patterns, while hydrogen fluoride-extracted silicatein leads to irregular clumps [91]. 

**Figure 8:**
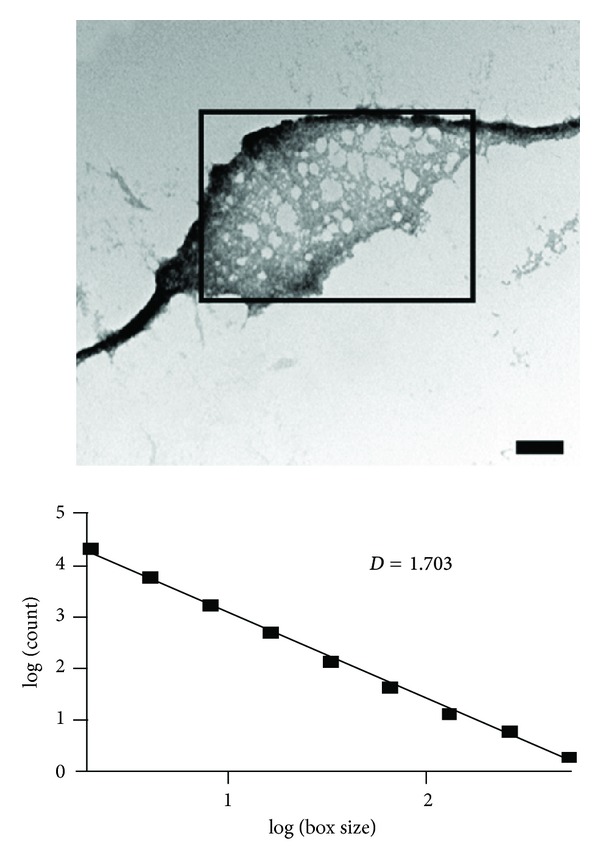
TEM images of the fractal structures formed by *T. aurantia* silicatein, initially depolymerized from spicules at pH 9 and then allowed to reassemble at low temperature. The fractal dimension of 1.7, obtained by counting the number of filled squares within boxes of increasing size, indicates formation of an elaborate self-assembly network by simple Brownian motion. Reproduced with permission from [100].

A key to the mode of association was provided by homology modeling of *T. aurantia* silicatein to cathepsin, which identifies five hydrophobic patches on the silicatein (but not cathepsin) surface [100], immediately suggesting a simple “sticky-patch” association. Native *T. aurantia* spicules may simply be dissociated by relatively mild alkali (pH 9) or chemical denaturants [90], leading to oligomeric structures which dissociate to even smaller oligomers in the presence of reducing agents, suggesting that the oligomers are stabilized intermolecularly by disulfide bonds [100]. Given a chance to reassemble at low temperature (but still at pH 9 to reduce unspecific association), the oligomers form complicated networks which nevertheless are an order of magnitude smaller than the native filaments and follow a fractal dimension of 1.7 [100]. This is interpreted to mean that they associate by Brownian motion in a random walk, and is most likely promoted by the anisotropic sticky surface (Figure 8). Interestingly, silicatein from the giant hexactinellid sponge *M. chuni* associate readily via dimers and trimers to higher filamentous structures but without evidence for intermolecular disulfide bond stabilization; they only show a fractal stage at the very beginning of their self-association [116] before they form long linear structures, and very rapidly become covered with cube-shaped silica particles which seem to grow by piling up. 

#### 4.2.3. Silicatein Assembly *In Vivo*

**Figure 9:**
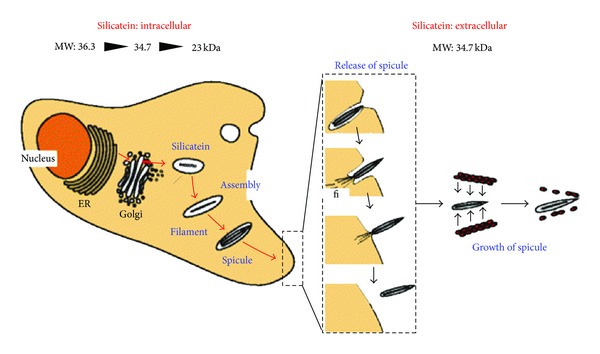
Model of spicule formation in *S. domuncula* This starts intracellularly by processing of silicatein to a mature and phosphorylated 23-kDa form, which assembles to axial filaments and starts to template silica deposition around itself. The spicules are extruded from the cells and mature in size in the extracellular stage with the help of the 34.7 kDa prosilicatein (lacking the signal peptide). No mature silicatein has so far been identified outside the cell. Reproduced with permission from [110].

**Figure 10:**
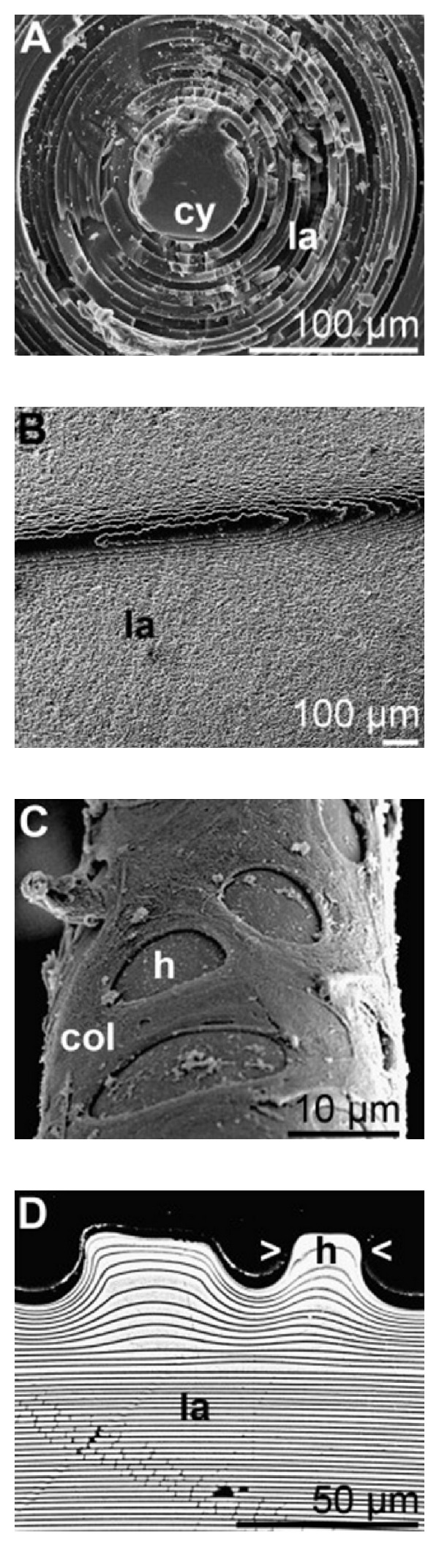
Scanning electron microscope analysis of *M. chuni* giant basal spicule. (A) Cross section of spicule shows concentric lamellar arrangement of silica layers (la) around the central axial cylinder (cy). (B) Longitudinal section shows highly folded arrangement of the lamellae (la). (C) Immature spicules are encased within a collagen (col) net with a regular pattern of holes (h) where silica material is visible. (D) Lamellae in the spicule form protrusions which neatly fit into these holes. Reproduced with permission from [111, 116].

Such studies reveal the fascinating complexity of aggregate structures even in simple *in vitro* systems. The question is whether these intriguing *in vitro *observations have *in vivo* relevance. The overarching purpose of silicatein is to build up spicule structures (Figure 9), and this occurs both by elongation at the tip of the filament as well as by apposition of silica concentrically around the filament [27]. *In vivo* the process is biologically constrained by a number of additional components, primarily galectin and collagen, which coexpress with silicatein *α* upon treatment with silicate [117]. Galectin aggregates in the presence of Ca^2+^ [117] and immune-gold electron microscopy studies indicate that it forms strings or nets that orient silicatein concentrically around the growing spicules, while collagen fibers are arranged in a highly ordered pattern around these spicules in the so-called extraspicular space [118] (Figure 10). This appears to be a universal pattern; similar conclusions have been reached for the Hexactinellid sponge *Monorhaphis chuni*, which synthesizes the largest biosilica structures on earth, forming giant basal spicules of length 3 meter and diameter 1.1 cm [119]! The concentric layers or lamellae in the spicules are generally more distinct in the Hexactinellids, making them excellent model systems to analyze how silicateins and other proteins organize silica. These spicules show 3 biosilica regions, namely, a central axial canal with the proteinaceous axial filament, surrounded by a bulky axial cylinder and up to several hundred lamellae, which gradually become thinner at the periphery [120, 121]. The axial filament itself is suggested to be linked to the inner surface of the growing spicule by membranous structures at the early stage of growth, but this connection is lost at a later stage as the axial filament contracts inside the canal, decreasing water concentration and promoting silica condensation [110]. The spicules are covered by a perforated collagen net [10] in dynamic equilibrium with the growing spicules: at early stages the collagen forms a tight corset around the inner layers but subsequently melts away as the spicule grows [116]. Serrations at the specular tips fit exquisitely into the perforated holes of the collagen net (Figure 10). 

Silicatein is found both in the axial filament and the lamella of *Monorhaphis* [10, 92, 122] and *C. meyeri *according to immunochemical analyses [111]; elegant nanoSIMS (secondary ion mass spectrometry) analyses of the lamellae highlight 3 sublamellae/2–6 *μ*m in width, each of which contains three cylindrical slats which are most likely delimited by silicatein or other proteins [120]. SAXS studies have shown that the basic unit of silica in spicules are nanospheres of diameter 2.8 nm [123]. These silica particles need to fuse or biosinter, and this probably happens in a hierarchical fashion from slats to sublamellae to lamellae and finally the axial cylinder. This process most likely requires silicatein and explains why the protein is integrated into the silica lamellae (where it also shows proteolytic activity [92]) as well as within the axial filament [120]. 

#### 4.2.4. Challenging Issues: Comparing The Self-Assembly of Different Silicateins with and without Helper Proteins

Clearly there is a very large diversity in the way that different silicateins organize themselves and this will likely impact how they interact with silica. The most accessible structure to mimic under simple *in vitro* conditions is probably the axial cylinder which is essentially made up of associated silicatein. It will no doubt be highly informative to carry out systematic comparisons of a diversity of different silicateins (in combination with galectins from different sponges) from the two classes of sponges to systematize different levels of structure, following the process by SAXS (particularly useful for the early stages of aggregation) and electron microscopy or Atomic Force Microscopy (for the complex architecture formed at later stages). 

Additional clues may come from the inclusion in these simple model systems of other proteins shown to be connected to silicatein, namely, the silintaphins. The 42.5 kDa silintaphin-1 (silicatein-*α* interactor with PH domain-1), identified from yeast two-hybrid screens with an *S. domuncula *library [47], colocalizes with silicatein in the axial filament as well as in the layers around it and appears to form a scaffold that can organize silicatein into filaments (rather than randomly organized aggregates) at ratios of 4 silicatein: 1 silintaphin-1. In this complex, silicatein also shows increased silicifying activity [124]. As a technological example, it helps silicatein form rod-like structures on Fe_2_O_3_ nanocrystallites [47]. Intriguingly, Silintaphin-1 contains so-called PEST stretches, which are hydrophilic regions enriched in disorder-promoting residues; these may promote limited proteolysis. Together with a highly repetitive C-terminal structure, a large number of hydroxyl-rich amino acids and lysine pairs (which could become modified by LCPAs, cfr. [77]), this gives silintaphin-1 many features in common with the silaffin proproteins from diatoms. Silintaphin-2, a smaller 15-kDa protein, was identified in a similar fashion and also colocalizes with silicatein inside and on the surface of spicules [38]. This protein has a large number of acidic and basic residues forming alternately positively and negatively charged clusters. It remains to be seen whether its ability to bind Ca^2+^ ions allows it also to bind silicate and then transport silicates to silicatein or whether its role is more indirect.

## 5. Biotechnological Applications of Silicifying Proteins and Peptides

The ability to control deposition of insoluble silica by silicate-binding proteins and peptides has been a technological dream in the biosilica community ever since the first identification of silicifying proteins. For a start, the spicules formed by this process have remarkably light-propagating properties which may be a substitute for a nervous system [125]: the spicular dimensions (core diameter and cladding layer thickness) are good for photonic band gaps in the infra-red, visible, and ultraviolet regions, leading to efficient single-mode waveguide and Bragg light propagation regimes [11]. In principle this can be used to develop new integrated optical elements with specific waveguide properties. Furthermore, spicules combine strength and flexibility through the combination of organic (protein) and inorganic (silica) material; a 3-point bending assay shows that the spicule's axial cylinder has high elasticity [10]. However, it still remains to be seen how these remarkable material properties can be translated industrially. Many interesting applications have been made of silaffins and silicateins, though we have yet to see their practical impact. Focused silica deposition has potential impact predominantly in the areas of bioencapsulation, nanopatterning and cell growth. We will deal with these in turn.

### 5.1. Bioencapsulation with Silaffin Peptides or Bacterially Displayed Silicatein

Bioencapsulation involves the encasing of biocatalysts, mainly enzymes, within a silica layer that will protect and stabilize the enzyme under adverse operating conditions while still allowing substrate access. Silica offers clear advantages compared to conventional polymer-based sol-gel encapsulation, which is limited by relatively harsh conditions to make the polymer medium [127], relatively long curing or hardening times (hours-days), limited porosity of the resulting gel and superficial binding/trapping of proteins. In contrast, silica remains mesoporous (2–50 nm pore size), forms rapidly (within minutes), shows much better weight protein binding capacity [128], and can be modified to coat surfaces with films of enzymes. The peptide of choice has been the 19-residue R5 peptide from *C. fusiformis *silaffin-1A. *In vitro*, the parent peptide silaffin-1A makes silica spheres in the size range 0.5–0.7 *μ*m with silica at for example, 12 silica: 1 silaffin molar ratios [50], particles of the same size are formed by the R5 peptide, though the process is pH dependent and does not work at low pH because of the lack of post-translational modification for the synthetically produced peptide as described above [50]. This is obviously not a problem for most technological applications.

There have been several successful reports of the encapsulation of the enzyme butyrylcholinesterase simply by mixing it with the R5 peptide and silicic acid (formed by the hydrolysis of tetramethyl orthosilicate) [66, 129]. The *in situ *deposition is so thorough (despite its rapidity [66]) that it leads to a loading capacity of up to 220 mg enzyme per g silica and proper trapping of the enzyme within silica rather than simple adsorption [129]. Importantly, the encapsulated enzyme is completely stable over 30 days at room temperature and survives freeze drying and elevated temperatures vastly better than the free enzyme, remaining active over 1000 column volumes [129]. The drawback is the very high concentration of R5 peptide required (10 mg/mL, corresponding to a molar ratio of 1 protein : 9000 R5). The obvious alternative is to fuse R5 directly to the protein. When this is done using GFP as a testing ground with N-terminal fusions of R5 and other silaffin-1A peptides, it turns out that the GFP fusion proteins are much more efficient at silicification at pH 7.0, requiring ~15 fold less protein for silicification compared to pure R5, and incorporating ~20-fold more silica per protein (molar ratio) than R5 [130]. Up to 85% of the protein can be immobilized [130]. Even higher efficiency of encapsulation (up to 99%) is obtained in a more recent report with R5 N-terminally fused to different enzymes produced recombinantly in *E. coli* [131], though the increase in stability is relatively modest [131]. It is not clear whether the proteins are all encased to the same extent and whether harsh conditions may “weed out” the least deeply encased proteins first: this would probably be revealed by multiexponential declines in enzyme activity over time. R5 peptide has also been fused to glucose oxidase to make an immobilized glucose sensor, though it remains unclear how well it works compared to other sensor constructs [132]. Surprisingly, the possible contributions of LCPAs to more efficient immobilization have not been explored.

A completely different approach has been taken with *T. aurantia* silicatein-*α*, namely, the encapsulation of living cells. Although it has been difficult to purify large amounts of active and folded silicatein from recombinant expression in *E. coli*, there is evidence that the protein is active when produced. Silicatein expression in *E. coli* is actually upregulated by silicic acid (even though it is under the control of the IPTG-responsive *lac* promoter), and this leads to a viscous cover of silica around the cells [134]. The silica deposition leads to cellular clumps up to 5 mm in size and with apparent fusion of the bacterial cell surfaces, though no adverse effect on growth kinetics. It is unclear whether silicatein is actually targeted to the surface of the cell or whether the silica diffuses in, so the mechanism of deposition remains to be elucidated. However, a more direct targeting of silicatein to the cell surface was carried out by inserting the gene for silicatein-*α* into the first extracellular loop of the *E. coli* outer membrane protein OmpA, the most common protein in the bacterial outer membrane. Silicatein shows a very general ability to condense many other oxides in addition to silicates, and this construct was able to form extracellular layered and nanocrystalline sheets of titanium phosphates from a water-soluble precursor [135], though there was also some evidence for nonspecific hydrolysis and deposition of titanium phosphate even in the absence of silicatein. The same cells could also promote the ring-opening condensation of L-lactide to form amorphous poly(L-lactide) [108]. Nevertheless it should be noticed in both cellular and *in vitro* contexts that the systems are limited by the small quantities of enzyme available, the passivation of the catalytic surface due to the deposition process and the competition from water in many of these reactions [108]. As of now, the cellular display of silicatein remains a good illustration of the principle of cell-based silica deposits. It is unclear whether this approach can be used to improve cellular performance in for example, metabolic bioreactors by coating cells in a protective “armour” to an extent that does not impede the desired metabolic processes, and whether there truly is going to be a protective effect; there is a great difference between encasing individual molecules in a silica phase as opposed to entire cells.

### 5.2. Nanopatterning

**Figure 11:**
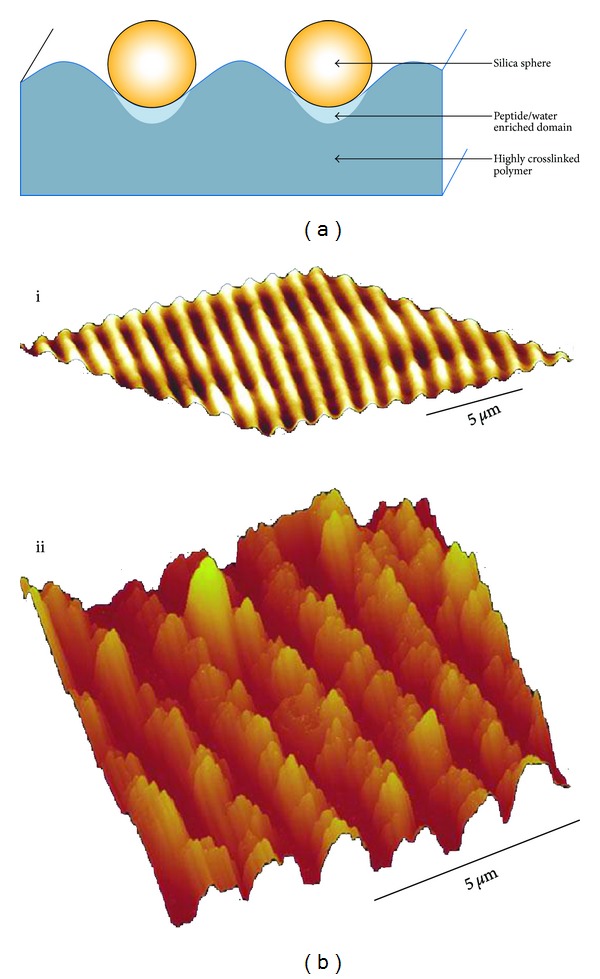
(a) Scheme of the cross-section of the hologram, indicating that the silaffin peptide R5 accumulates in in the polymer troughs. (b) AFM images of the polymer (i) before and (ii) after silification. The peaks in (i) correspond to the troughs in (ii), where the most prominent features are the silica deposits. Reproduced with permission from [126].

Nanopatterning with silica holds obvious advantages due to the refractive properties of silica. An impressive early example is the use of the R5 peptide to make nanopatterns in acrylate structures; when the R5 peptide was mixed with monomeric light-sensitive precursors and exposed to laser light in a holographic arrangement, the R5 phase-separated with the 1.33 *μ*m periodicity of the laser beam [126]. Subsequent addition of tetrahydroxysilane led to formation of silica spheres in the troughs with a highly regular hologram periodicity and a ~50x increase in diffraction efficiency (Figure 11). Dimensionality was increased even further by using silaffin-1A to biosculpt silica nanoparticles from tetramethylorthosilicate under linear shear flow (given that variations in shear flow can lead to highly layered structures [136]), leading to 3D interwoven microfilamentary silica structures [137]. If these structures are exposed to gaseous magnesium, Si is replaced by Mg leading to nanocrystalline MgO microfilaments with the original shape retained. However, it remains to be seen how much better these work than simple (and much less expensive) polypeptides; for example poly-L-lysine forms hexagons or spheres of silica if left undisturbed, but linear flow or electric fields led to complex three dimensional structures including petals and fibrils, all made of fused spherical particles but with periodic voids [138]. The same effect of external influences has been seen in the structures of silica deposits with silicatein mutants from directed evolution, where agitation leads to dispersed nanoparticles while stagnant incubation leads to more sheet-like structures [112]. This phenomenon is also known from the field of protein aggregation, where for example vigorous shaking can lead to fibrils with a very different structure from that of fibrils formed under quiescent conditions [139, 140]. The difference likely reflects how shear forces in solution affect the pathways of nucleus formation and coalescence.

**Figure 12:**
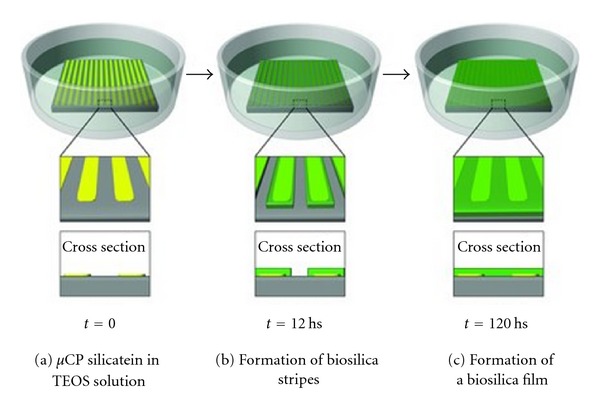
Formation of biosilica layers by controlling imprinting of silicatein. (a) Silicatein is microcontact printed onto the surface and the silica precursor TEOS is added. (b) Initially silica only covers the silicatein strips. (c) Over time the strips fuse to form a continuous layer or film. Reproduced with permission from [133].

There are even more reports with silicatein than silaffin for nanotechnological applications, not least because of silicatein's versatility as a general promoter of oxide condensation. A particular advantage with immobilized silicatein is that clustering of silicatein may lead to “composite nucleation sites” which can lower the activation barrier to heterogeneous (solid-solution) nucleation [106]. At the same time, the relatively low catalytic efficiency of silicatein means that the material produced at the surface has sufficient time to rearrange structurally to states which are the most stable (but not necessarily kinetically the most accessible) at the nano- or microscale [108]. *S. domuncula* His-tagged silicatein can be immobilized on gold surfaces modified with alkanethiol [141] or cysteamine/reactive ester polymers [106] coupled to a nitrilo-triacetate group and the reaction followed by surface plasmon resonance. Using the conventional TEOS precursor, silica nanospheres form on the surface [141] while layered nanoparticles of titania (TiO_2_) and zirconia (ZrO_2_) form with other precursors. The drawback with this approach is the relatively large number of steps required to generate appropriately bound NTA molecules and the lack of control over the thickness and roughness of the silica films. These two problems have been reduced in a recent report in which cystamine or cysteamine or linked to the gold surface, then functionalized with glutaraldehyde to allow silicatein to be subsequently immobilized before TMOS (tetramethylorthosilicate) is added [142]. Layer thickness, roughness, and water contact angle can be controlled by varying the amount of silicatein adsorbed on the layer and the time of exposure. The importance of the time dimension is also highlighted in a study where silicatein is microcontact printed in strips on the surface by conventional optical lithography (physisorption), followed by addition of TEOS [133]. At early stages, silica only forms at the strips where silicatein is present and only gradually merges to a continuous layer (Figure 12). It is very likely that the sub-*μ*m particles aggregate to form the layers, just as is seen in the biosintering processes *in vivo* [120]. Nevertheless, we are still missing a systematic comparison of different silicifying biomolecules—from silaffins with and without LCPAs to silicateins and Cys-Lys block copolymers—to establish which of these holds the greatest potential for efficient and spatially controlled silica depositioning.

In the field of organic Si-based synthesis, silicatein has shown some interesting properties. Purified filaments of silicatein can be converted to catalysts for processes as diverse as Heck reactions or binding of SO_2_ gas; this occurs by incubating them with alkoxy silanes with metallated pincer complexes, integrating the pincer into silica [143]. In the first description of an enzymatically enhanced organometallic condensation, recombinant silicatein (modestly—only 2-fold) catalyzes the condensation of alkoxy silanes at neutral pH and ambient temperature to yield silicones like straight-chained dimethylsiloxane [144]. It seems that we are only limited by our imagination (and feasible scaling-up) in terms of finding appropriate applications.

### 5.3. Silicatein, Silaffins, and Cell growth

Both silaffins and silicatein show promise in stimulating growth of bone cells (osteocytes). Their precursor mesenchymal stem cells adhere better to roughened mineral surfaces [145]. Exposure of human osteosarcoma SaOS-2 cells to biosilica increases expression of structural molecules of the enamel matrix as well as the level of production of hydroxyapatite, the inorganic calcium-phosphate component of bone [146] and enhances cell proliferation more than growth on inorganic calcium phosphate surfaces [147]. Silica is already used as the main constituent of many biocompatible porous scaffold materials [148]. Since silica can be seen as a mimic of hydroxyapatite, controlled deposition of silica can be of great potential use in modulating the rate and extent of tissue regeneration for use in bone regeneration and tooth reconstruction *in vivo*. This has been exploited in several contexts. Focused deposition of silica via controlled immobilization of silicifying proteins is obviously critical. 

#### 5.3.1. Deposition with Silicatein

Silicatein has been deposited onto culture plates by simple physisorption [149] followed by the addition of TEOS to deposit biosilica, leading to a marked increase in the formation of calcium phosphate nodules by human osteosarcoma SaOS-2 cells. However, an even more ingenious approach has been to add an 8-Glu tag to the N-terminus of the protein which confers binding to hydroxyapatite, promoting the formation of biosilica on synthetic hydroxyapatite nanofibrils and dental hydroxyapatite upon addition of biosilica precursor [150]. Such a “smart glue” can potentially seal surface defects and dentinal tubules to reduce risk of tooth decay and dental hypersensitivity. It would be interesting to explore whether limited expression of silicatein on these cells and subsequent depositioning of biosilica directly on the cell surface (cfr. the bacterial display system in *E. coli* [134]) could stimulate osteoblast mineralization even further, though there is an obvious disadvantage in the continuous expression of silicatein and potential for unwanted biosilica formation unless the precursor supply is removed.

#### 5.3.2. Deposition with Silaffin

For silaffin this has involved fusing (genetically) the R5 peptide with peptides that can self-assemble into *β*-sheet-rich (and biologically degradable) structures, either the consensus repeat of the major ampullate spidroin protein 1 (MaSp1) from spider dragline [151] or a hydrophobic-polar EAK_1_ polymer (AEAEAKAKAEAEAKAK) [152]. The R5-EAK_1_ construct does not appear to have any obvious advantages other than altering the morphology of the deposits (which in itself can be useful if tailored). However, the R5-MaSp1 construct deposits silica with around 20-fold greater efficiency (per R5 molecule) than free R5 [151]. Exploiting the self-assembling properties of MaSp1, it is possible to assemble silica on electrospun fusion protein fibers or even carry out spinning and silicification concomitantly to get more nonuniform coating [151]. The R5-MaSp1 fusion has subsequently been demonstrated to form three different types of silk-silica surfaces [153]. These surfaces can be controlled by altering the concentration of the silk films, since an increase of the protein concentration leads to much denser silica networks and silicification is enhanced by inclusion of an N-terminal His-tag; furthermore, the use of glycerol aids dispersion of the silica deposits. Particularly the high-concentration silk-film with a dense porous silica network enhances osteogenesis of human mesenchymal stem cells, as can be seen from the upregulation of osteogenic gene markers [153].

### 5.4. Challenging Issues: Silaffins versus Silicateins?

What is lacking at the moment is a direct comparison of how well silaffin- and silicatein-mediated silica deposits perform in all these contexts. For example, could silicatein benefit from being coupled to the MaSp1 or EAK_1_ sequences in terms of efficiency and usefulness of silica deposition? These applications are fascinating and compelling but they have yet to provide more insight into the underlying mechanisms of silicification and an understanding of how this may be coupled to applications.

## 6. Conclusion

In the present paper, I have attempted to juxtapose two different systems for biosilicification, those of the diatoms and the sponges. While the outcome is spectacularly different—comparing the unicellular *μ*m-sized diatoms with the macroscopic features of the sponges—both strategies involve proteins which combine self-assembling properties with the ability to interact with silica precursors through both hydrogen bonding and electrostatic interactions. The diatom system provides the potential for an almost infinite variety through the use of small silaffin peptides with different levels of post-translational modification (primarily of lysines and serines) combined with either free or covalently attached long-chain polyamines. For sponges, the far less heavily modified silicateins vary in their self-assembling properties and the coexistence of closely related isoforms. Both systems involve ancillary proteins which can promote or inhibit various types of assembly. The challenge now is to apply techniques used to analyze formation of complex systems such as SAXS together with atomic-level structural information from, for example, solid state NMR and combine this insight with the systems-level information from genomic analyses to include appropriate components in representative model systems. Biosilicifying organisms will continue to serve as inspiring examples of nature's ingenious way of solving biological problems with nanotechnological tools—using dilute precursor molecules and carrying out the “manufacturing” at ambient temperatures and pressure and neutral pH.

## Figures and Tables

**Table 1:** 

	Diatoms	Sponges
Silica structure	Cell wall	Body skeleton (multilamellar spicules of varying sizes)
Silica storage organelle	Silicon deposition vesicle with silicalemma	Silicasomes
Major silicifying protein	Small silaffin peptides (mainly 2.5–3 kDa)	Large silicatein protein (36 kDa in *S. domuncula*), homologous to cathepsin.
Precursor *in vitro *	Silicic acid (occurs naturally though orthosilicate has never been isolated *in vivo*)	Silicon alkoxides such as tetraethoxysilane (not identified *in vivo*)
Mechanism of silicification	General stimulation of polycondensation by electrostatic interactions	Catalysis of condensation with well-defined catalytic residues
Post-translational modifications	Lys hydroxylation, methylation, long chain amines Ser phosphorylation (kinase identified) Hydroxy(phospho)prolines Also glycosylation and sulfation	Phosphorylation is required for silicatein oligomerization.
Long chain polyamines	Covalently attached to silaffins and in some cases free in solution. Play a major role in silicification.	No LCPAs (except for *Axinyssa aculeate* where they can deposit silica and are associated with spicules [77])
Additional protein components	Cingulins, silacidins	Collagen, galectin, and silintaphins
Protein scaffold assembly	Unclear how the silaffins assemble *in vivo *	Silicatein forms an axial filament at the core of the spicules and coats the spicule surface to promote growth by apposition
